# Fracture Resistance of Teeth Restored with Anatomic, Universal 2-Piece, and CAD/CAM Milled Glass Fiber Posts in Various Weakened Root Conditions

**DOI:** 10.4317/jced.63165

**Published:** 2025-10-01

**Authors:** Rodrigo Stadler Alessi, Giovana Mongruel Gomes, João Carlos Gomes

**Affiliations:** 1 DDS, MS, PhD student, Postgraduate Program in Restorative Dentistry, Department of Restorative Dentistry, State University of Ponta Grossa (UEPG), Ponta Grossa, Paraná, Brazil; 2 DDS, MS, PhD, Professor, Department of Restorative Dentistry, School of Dentistry, State University of Ponta Grossa (UEPG), Ponta Grossa, Paraná, Brazil

## Abstract

**Background:**

This study aimed to evaluate the performance of CAD/CAM milled, universal 2-piece and anatomical fiber posts in comparison with prefabricated fiber posts in root canals with varying degrees of structural compromise, using fracture resistance (FR) testing and failure mode analysis.

**Material and Methods:**

Seventy mandibular premolars were selected, trimmed 2 mm above the cementoenamel junction, and subjected to endodontic treatment. The specimens were divided into seven groups based on the type of restorative approach (prefabricated, anatomical, CAD/CAM milled, and universal 2-piece fiber posts) and the extent of root weakening (no weakened, moderately weakened, and severely weakened). RelyX U200 cement was used for post luting, followed by the fabrication and cementation of metal-free crowns. Failure rates and patterns were evaluated, with statistical analysis performed using Student’s t-test, two-way ANOVA, Tukey’s post hoc test, and chi-square tests to compare failure mode frequencies.

**Results:**

The findings revealed that CAD/CAM milled and universal 2-piece fiber posts demonstrated significantly higher fracture resistance compared to anatomic fiber posts. Moderately weakened (MW) specimens outperformed severely weakened (SW) ones. All experimental groups exhibited fracture resistance comparable to the control group, except for the MW-MFP group, which exceeded the control. The majority of fractures were repairable, and no significant association was observed between failure mode and the type of restorative approach or the degree of root weakening.

**Conclusions:**

CAD/CAM milled and universal 2-piece fiber posts offer strong fracture resistance and predominantly repairable failure modes, making them suiTable alternatives for restoring weakened roots.

** Key words:**Resin Cements, CAD-CAM, Post and Core Technique, Root Canal Preparation.

## Introduction

Intra-radicular retainers are widely used in dental rehabilitation for root canal-treated teeth with substantial coronal structure loss. These retainers are crucial for enhancing the retention of core build-up materials and supporting subsequent restorative procedures [1]. Historically, custom-cast metal posts and cores have been a preferred choice, as supported by numerous studies [1]. These posts provide improved compatibility with root walls without requiring alterations to the canal’s geometry [2]. However, the high modulus of elasticity of cast metal posts can generate a wedging effect under occlusal forces, potentially leading to severe root fractures [2].

Glass fiber posts (GFPs) have emerged as a viable alternative due to their dentin-like physical properties, resistance to corrosion, and modulus of elasticity similar to dentin. These characteristics enhance stress distribution and reduce the risk of root fractures [3,4]. Despite these benefits, precise fitting of GFPs in larger root canals remains a concern [5]. Inadequate adaptation can result in thick resin cement layers, increasing the likelihood of voids or bubbles and, consequently, the risk of failure [6].

Several strategies have been proposed to minimize resin cement thickness, including the use of accessory fiber posts [7], anatomical posts fabricated directly or indirectly [7-9], and root reinforcement with restorative materials [8]. The direct anatomical post technique, which involves relining prefabricated fiber posts with composite resin, improves post adaptation to the root canal, resulting in thinner cement layers, enhanced retention, and better mechanical properties than accessory posts or root reinforcement techniques [8]. However, this approach poses challenges such as void formation, polymerization shrinkage of the resin composite, and technique sensitivity [8].

It has been demonstrated that prefabricated posts with close adaptation to the canal yield higher bond strength compared to relined posts [10]. Moreover, both closely fitting and relined posts exhibit superior bond strength compared to poorly adapted prefabricated posts in flared canals [10]. Advances in CAD/CAM technology now offer innovative solutions for customized GFP fabrication, allowing the creation of post-and-core units as a single component [11,12]. This eliminates the need for a composite resin core, reducing potential failure points at the material interface [11]. Additionally, CAD/CAM-milled posts provide superior adaptation to canal walls, resulting in thinner cement layers [12-15].

A novel universal two-piece GFP system (Splendor Universal; Angelus) has recently been introduced. This system includes a single-size post adapTable to narrow, medium, and wide root canals through a conical sleeve and a thin post. Its design allows for incremental adaptation from the apical to cervical third, enabling micromechanical adhesion, mechanical retention, and more conservative root preparation [16,17].

This study aims to assess the fracture resistance and failure modes of endodontically treated teeth restored using GFPs fabricated via CAD/CAM technology and the Splendor Universal two-piece system, comparing them with traditional methods such as conventional prefabricated and anatomical fiber posts. The null hypothesis tested is that fracture resistance and failure modes are unaffected by the restorative strategy (fiber post type) or the degree of root weakening.

## Material and Methods

This prospective study was approved by the Research Ethics Committee of Ponta Grossa State University (protocol no. 56948022.7.0000.0105). Seventy intact human mandibular premolars were selected based on stringent inclusion criteria, requiring a minimum root length of 14 mm and the presence of a single root canal. Teeth with prior endodontic treatment, caries, root fractures, or resorption were excluded. Radiographic analysis was conducted to confirm compliance with these criteria.

The sample size for the fracture resistance test was established using data from a pilot study (mean 1041.2 MPa, standard deviation 160.3 MPa), employing the formula: *n* = Z2xs2/e2.

where Z = 1.96, acceptable error (e) = 10% of the mean, and s = 160,3 (standard deviation). Thus, a minimum sample size of 9.11 was calculated, leading to the random selection of 10 teeth per group.

- Specimen Preparation 

Endodontic Treatment 

The selected teeth were sectioned perpendicularly 2 mm above the cementoenamel junction (CEJ) using a diamond disc mounted on an ISOMET 1000 cutting machine (Buehler, Lake Bluff, IL, USA) operating at 300 rpm with continuous water irrigation. This facilitated access to the root canal and ensured precise root length measurements, which were confirmed with a millimeter ruler.

Root canal instrumentation was performed using rotary files up to the #F3 tip (Protaper Ultimate, Dentsply Maillefer, Ballaigues, Switzerland). During instrumentation, canals were irrigated with 1 mL of 2.5% sodium hypochlorite solution. Following this, saline irrigation and final cleansing with 17% ethylenediaminetetraacetic acid (EDTA) (Fórmula e Ação, São Paulo, SP, Brazil) were performed for three minutes, with solution replacement every minute. The canals were then rinsed with saline and dried with absorbent paper points (Dentsply Maillefer, Petrópolis, RJ, Brazil).

Obturation was carried out in the apical 4 mm of the canal using the initial step of the vertical compaction technique by Schilder (1967) [18]. Heated gutta-percha cones (Dentsply Maillefer, Petrópolis, RJ, Brazil) and an epoxy resin-based sealer (AH Plus, Dentsply, Petrópolis, RJ, Brazil) were used, following the manufacturer’s guidelines. Periapical radiographs (Kodak Ultra, Eastman Kodak, NY, USA) were taken to confirm complete obturation within the 4 mm apical region. The canal orifices were sealed with conventional glass ionomer cement (GIC) (Vitro Fil, DFL, Rio de Janeiro, RJ, Brazil).

- Post Space Preparation 

After one week of storage at 37 ± 1 ºC in high relative humidity (Eppendorf tubes with gauze soaked in distilled water), the post spaces were prepared for the insertion of intraradicular posts. The preparation measured 12 mm depth, with a 4 mm length of obturation material retained. The root canals were initially prepared with #2, 3, and 4 Largo type drills (Jota, Florianópolis, SC, Brazil).

- Experimental Groups 

**Figure 1:**
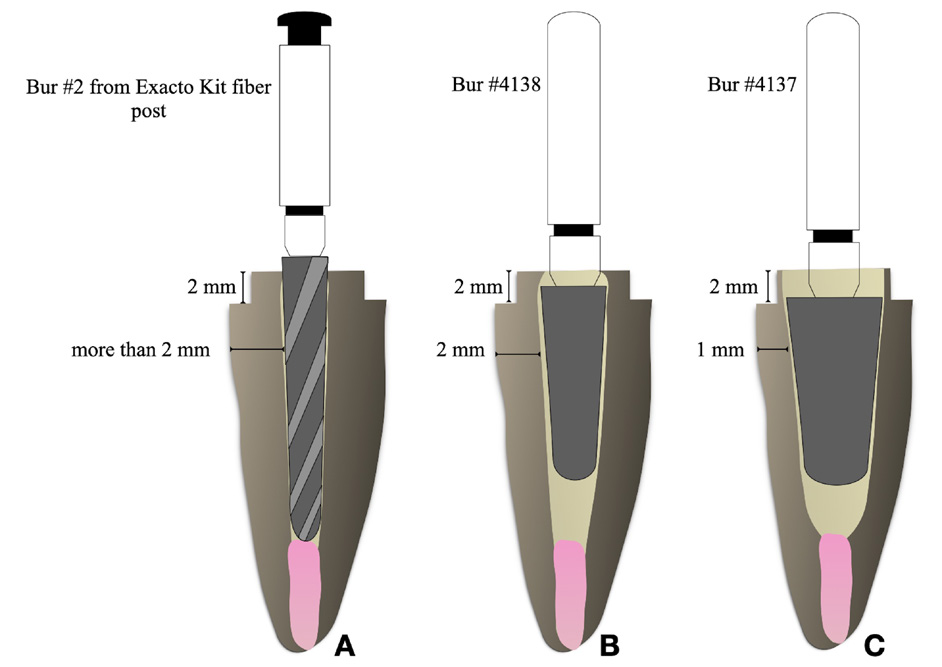
Schematic representation of roots fragilization. (A) Non-Weakened; (B) Moderate Weakened; (C) Severe Weakened.

**Figure 2:**
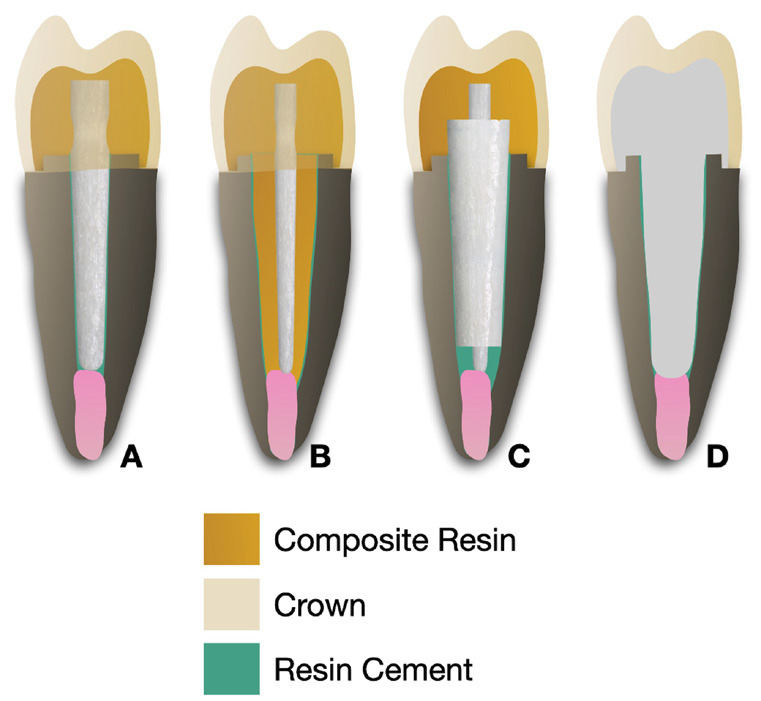
Schematic representation of the restoratives strategies. (A) Prefabricated fiber post; (B) Anatomical fiber post; (C) Universal 2-piece fiber post; (D) CAD/CAM milled fiber post.

**Figure 3:**
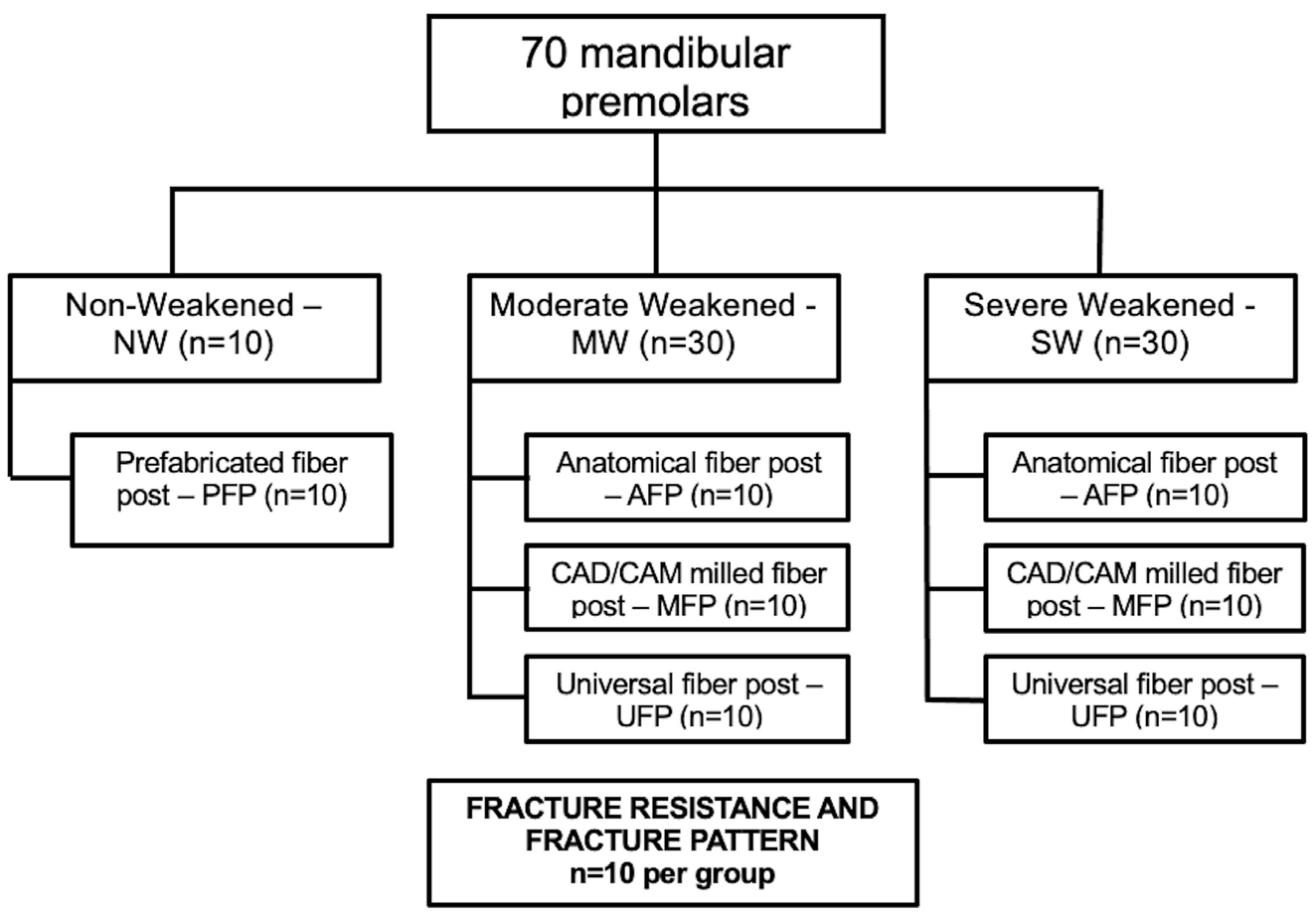
Study design flow chart. Abbreviations: CAD/CAM, computer-aided design, and computer-aided manufacturing.

The roots (*n*=70) were randomly divided into 7 experimental groups based on the following factors: weakening protocol – non-weakened (NW), moderately weakened (MW), or severely weakened (SW); and restorative approach – prefabricated fiber post (PFP), anatomical fiber post (AFP), universal 2-piece fiber post (UFP), and CAD/CAM milled fiber post (MFP) (Figs. 1,2). The description of the groups can be found in  Table 1.  Table 2 contains the description of the glass fiber retainers and the restorative materials utilized in the investigation. A flowchart illustrating the study design can be found in Fig. 3.

- Weakened Protocol

Non-weakened (NW) – control group: roots remained intact and were solely prepared using the appropriate drill for the glass fiber post (Exacto #2; Angelus, Londrina, PR, Brazil) attached to the micromotor, exhibiting a residual dentinal wall thickness exceeding 2 mm in the coronal region (Fig. 1).

Moderately weakened (MW): A conical diamond-tipped bur with a rounded top, specifically #4138 (ø 1.8 mm) (KG Sorensen, Barueri, SP, Brazil), was employed to widen the root canals, yielding a residual dentinal wall thickness of 2 mm in the coronal region (Fig. 1).

Severely weakened (SW): A conical diamond-tipped bur with a rounded top # 4137 (ø 2.5 mm) (KG Sorensen, Barueri, SP, Brazil) was employed to extend the root canals for root fragilization, yielding a residual dentinal wall thickness of 1 mm in the coronal region (Fig. 1).

In all instances of root canal preparation, the root widening was conducted uniformly to a depth of 12 mm, with a diameter matching to that of the individual active tip. The remaining dentine measures were verified using a digital caliper (Digimatic Caliper, Mitutoyo, Japan). A new diamond bur was utilized for every three roots. Upon completion of this stage, the roots were examined for the existence of cracks, and those exhibiting cracks were excluded from the study. In the control group, the roots remained intact.

Subsequently, all root canals were re-prepared using the bur designated for fiber post n° 2 (Exacto, Angelus, Londrina, PR, Brazil). The root canals were then irrigated with 10 mL of distilled water and air-dried for 5 seconds from a distance of 3 cm, followed by the insertion of two absorbent paper points.

- Restorative Approach

For Prefabricated fiber post (PFP) group, fiberglass posts (Exacto No. 2, Angelus, Londrina, PR, Brazil) were cemented into intact roots simulating well-adapted fiber posts to the root canals.

In the Anatomical Fiber Post (AFP) groups, fiberglass posts (Exacto No. 0.5, Angelus, Londrina, PR, Brazil) were cleaned with 70% alcohol for 5 seconds, following the manufacturer’s recommendations. A universal adhesive system (Single Bond Universal, 3M ESPE, Sumaré, SP, Brazil) was applied in two layers using the self-etch technique. Each layer was applied for 20 seconds, air-dried for 5 seconds, and light-cured for 10 seconds. The root canals were lubricated with a water-soluble gel (KY lubricating gel, Johnson & Johnson, São José dos Campos, SP, Brazil). The prefabricated post was coated with a regular viscosity bulk-fill composite resin (Filtek One Bulk Fill, shade A1, 3M ESPE, Sumaré, SP, Brazil) and inserted into the root canal, ensuring correct positioning relative to the vestibular region and the coronal remnant. The assembly was inserted and removed twice to eliminate excess composite resin before being light-cured for 20 seconds while positioned inside the canal. The post was then removed, and an additional 20-second light curing was performed on all surfaces of the rebasement. The final anatomical post was assessed for complete adaptation within its respective root canal. Before cementation, both the root canals and anatomical posts were thoroughly washed with water and dried. 

The Universal 2-piece fiber post (UFP) system comprises a universal cylindrical post paired with a tapered sleeve made from the same material. The sleeve is designed with a decreasing thickness from the cervical to the apical end, forming a tapered shape and incorporating a longitudinal slot to enhance its adaptability between the post and the canal walls. Posts were positioned in the prepared root canals, and sleeves were inserted over them, ensuring correct apical alignment.

To produce CAD/CAM milled fiber posts (MFP), the post space was isolated using a water-based gel. A prefabricated acrylic resin pin (Pinjet, Angelus) coated with acrylic resin (Duralay-Reliance Dental, Alsip, IL, USA) was used to shape the post. The acrylic resin posts were then digitized using a digital scanner (TRIOS, 3Shape, Copenhagen, Denmark), and computer-aided design (CAD) was used to create posts tailored to the dimensions of the root canals. These designs were fabricated by milling glass fiber discs (Fiber Cad - Post & Core, Angelus Indústria de Produtos Odontológicos S/A, Londrina, PR, Brazil) using an industrial computer-aided manufacturing (CAM) system (Ceramill Motion 2, Amann Girrbach, Curitiba, PR, Brazil).

- Luting Procedures

Before cementation, anatomical posts and universal 2-piece posts were transversely sectioned to a length of 15 mm using a double-sided diamond disc (KG Sorensen) under continuous water cooling. This ensured that 12 mm of the post extended into the root working length, while the remaining 3 mm standardized the light-curing distance and confirmed the proper seating of the posts within the prepared root canals. The glass fiber posts, along with the sleeves in the UFP group, were cleaned with 70% alcohol for 5 seconds, dried with an air stream, and treated with silane (Monobond, Ivoclar Vivadent, Schaan, Liechtenstein).

All posts and cores (n = 70) were cemented using self-adhesive dual-cure resin cement (RelyX U200; 3M ESPE, St. Paul, MN, USA) according to the manufacturer’s instructions. For the PFP, AFP, and MFP groups, posts were inserted and adjusted within the canals, while in the UFP group, a universal cylindrical post was placed first, followed by the gradual insertion of a tapered sleeve until resistance was encountered, ensuring proper fit between the cervical and middle thirds. Posts were seated with consistent digital pressure and light-cured for 60 seconds.

- Preparation and Cementation of Indirect Crown

The preparation and cementation of indirect crowns are made based on previous study [20]. The core build-up involved preparing a master ceramic crown with a uniform thickness of 1 mm and a 2-mm occlusal reduction on a typodont (Pronew Odonto). Standardized preparation was performed using #3216 and #4138 diamond burs (KG Sorensen, Barueri) with a high-speed handpiece. Templates were fabricated using a 1-mm acetate sheet (Acetate Plate; Bio-Art) in a vacuum thermomolding apparatus (PlastVac P7; Bio-Art). An adhesive system (Single Bond Universal; 3M ESPE) was applied in two layers to the dentin and post, followed by light curing for 20 seconds. Composite resin cores were fabricated using regular viscosity bulk-fill composite resin (Filtek One Bulk Fill, shade A2, 3M ESPE). The resin was placed in a single 5-mm increment and light-cured for 20 seconds with the aid of an acetate template. A 1-mm-deep chamfer with rounded axial and occlusal-axial angles was then prepared, ensuring a continuous and well-defined cervical finish line.

Finishing and polishing were conducted with the Diamond Pro disc system (Diamond Pro, FGM, Joinville, SC, Brazil) in a sequential manner (coarse to extra fine) for 20 seconds per disc. Preparations were scanned using an intraoral scanner (3Shape TRIOS, 3Shape), and STL files were exported to design software (Ceramill Mind, Amann Girrbach) to standardize the buccal cusp height at 1.5 mm. Crowns were milled from 5 mol% yttria-tetragonal zirconia discs (5Y-TZP, Ceramill Zolid FX, Amann Girrbach) using a Ceramill Motion 2 machine and sintered in a Ceramill Therm furnace. The sintering cycle included a gradual temperature increase to 1450°C with a dwell time of 2 hours, followed by controlled cooling to room temperature over 5 hours.

After fabrication, the crowns were tested and adjusted. The internal surface was air-abraded with 50 μm aluminum oxide particles at 2 bar pressure for 10 seconds, treated with silane (Monobond N, Ivoclar Vivadent), and air-dried. The enamel surfaces were etched with 37% phosphoric acid (Condac 37%, FGM) for 15 seconds, then rinsed and dried with an air stream. Resin cement (RelyX U200, 3M ESPE) was applied inside the crown using a Centrix Precision syringe (Maquira) and seated under pressure. Light curing was performed for 20 seconds on each surface to ensure proper polymerization.

All photoactivation procedures used a light source with an irradiance of 1,400 W/cm² (Valo Cordless, Ultradent, St. Jordan, UT, USA), monitored by a digital radiometer (LM 1, Woodpecker, Guilin, China).

- Periodontal Ligament Simulation 

According to Taques *et al*. (2023) [11], to simulate periodontal ligament conditions, a 0.3-mm-thick wax coating (Lysanda Produtos Odontológicos) was applied to the root surfaces, extending 3 mm below the cementoenamel junction (CEJ). The wax layer was built incrementally using a gas lamp and sculpting tools, then measured with a digital caliper (Mitutoyo, precision 0.01 mm). Roots were embedded in acrylic resin (Clássico, Campo Limpo Paulista, SP, Brazil) within polyvinyl chloride (PVC) cylinders (Tigre, Joinville, SC, Brazil). After polymerization, the wax was removed, and the voids were filled with polyether impression material (Impregum Soft, 3M ESPE). Roots were reinserted, and excess material was trimmed with a scalpel, resulting in a standardized 0.3-mm periodontal ligament.

- Analysis of Fracture Strength Under Compression

Specimens with crowns and periodontal ligament simulation underwent fracture resistance testing. Using a universal testing machine (Shimadzu AG-I, Columbia, MD, USA), the specimens were positioned at a 150° angle to replicate occlusal forces. A load of 3000 N was applied at 0.5 mm/min using a knife-edge tip (10 mm wide, 5 mm thick) until fracture or displacement occurred [11]. Data were recorded in newtons.

- Evaluation of the Fracture Pattern 

According to Taques *et al*. (2023) [11], after the fracture resistance assessment, the roots were removed from the PVC tubes, the artificial periodontal ligament was removed, and the fracture patterns were analyzed, categorizing them based on the degree of tooth destruction: reparable (would allow repair)— displacement of the crown, post fracture, or root fracture above the root cervical level that would allow a new restoration; and irreparable (nonrestorable)— fracture below the root cervical third, vertical or oblique fracture, or horizontal fracture in the middle and apical thirds, which condemn the tooth to extraction.

The roots were examined at a magnification of 7.5× with a stereomicroscope (Discovery V20, Zeiss, Oberkochen, Germany), and the failure mode was also classified as: type I: fracture/displacement of the crown and or post; type II: a fracture in the cervical third of the root; type III: a fracture up to the middle third of the root; type IV: one or multiple vertical fractures, beyond the middle third of the root. Types I and II were considered repairable, allowing restoration, while types III and IV were considered irreparable or catastrophic fractures (adapted from Junqueira *et al*. [19]).

- Statistical Analysis 

The fracture resistance values were submitted to statistical analysis, using the Student’s t-test to compare each group with the control group, the parametric test ANOVA (two-way), and Tukey’s post hoc test (α = 0,05) to compare the experimental groups with each other.

The frequency of failure types was compared using the chi-square test. All calculations were performed using Sigma Plot 11 statistical software (Systat Software, San Jose, CA, USA). The failure modes were analyzed qualitatively only.

## Results

The two-way ANOVA revealed no statistically significant interaction (*p* > 0.05) among the weakening protocols (moderately or severily) and restorative approachs (relined, universal, or milled fiber post); however, it was significant for the main factors of weakening protocol (*p* < 0.05) and restorative approachs (*p* < 0.001).

 Table 3 summarizes the mean and standard deviation of fracture resistance (N) in relation to the compromised protocol and restorative approach. The decreased protocol exhibited the lowest fracture resistance values in the SW group, demonstrating a significant difference when compared to the MW group (*p* < 0.05). In terms of restorative technique, MFP and UFP exhibited comparable fracture resistance values, both of which were significantly greater than those of AFP (*p* < 0.001).

The Student’s t-test for independent samples indicated a statistically significant difference between MW-MFP and the NW-PFP [control group] ( Table 4). The MW-MFP exhibited superior fracture resistance compared to the control group (*p* = 0.002). Other groups had no statistically significant change from the control group.

The chi-square test indicated no significant correlation between failure mode and restorative approach (*p*=0.161), nor between failure mode and weakened protocol (*p*=0.842) following the fracture resistance test ( Table 5).

**Figure 4:**
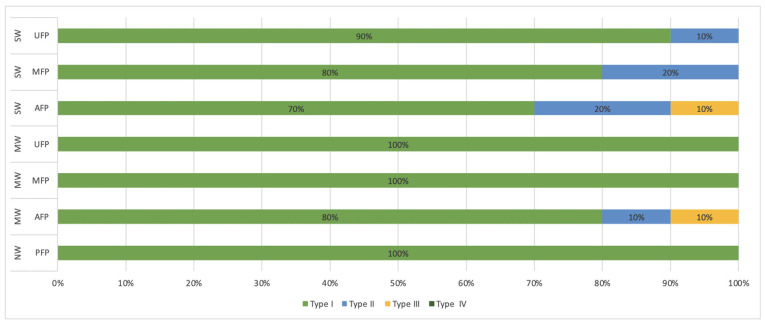
Frequency (%) of failure mode types in all groups. (SW: Severe Weakened; MW: Moderate Weakened; NW: Non-Weakened; UFP: Universal Fiber Post; MFP: CAD/CAM Milled Fiber Post; AFP: Anatomical Fiber Post; PFP: Prefabricated Fiber Post).

Figure 4 illustrates the prevalence of failure kinds within each group. Type I fractures predominated in all groups. Fracture type II was identified in the MW-AFP and SW-UFP groups, each representing 10% of occurrences, as well as in the SW-AFP and SW-MFP groups, both demonstrating a frequency of 20%. Type III fractures were observed in the SW-AFP and MW-AFP groups, each at a prevalence of 10%. Significantly, none of the groups demonstrated type IV failure.

## Discussion

This study investigated the fracture resistance of weakened roots restored with anatomical (AFP), universal 2-piece (UFP), and CAD/CAM-milled fiber posts (MFP). The results led to the rejection of the null hypothesis for the main factors: weakening protocol (*p* < 0.05) and restorative approach (*p* < 0.05), indicating significant differences. However, the interaction between weakening protocol and restorative approach showed no statistically significant difference (*p* > 0.05).

When each experimental group was compared individually to the control group (NW), the Student’s t-test revealed that both AFP and UFP groups demonstrated comparable fracture resistance, irrespective of the degree of root weakening. Notably, MFP exhibited significantly higher fracture resistance under moderate weakening conditions and comparable resistance under severe weakening conditions. These findings align with the observations of Penteado *et al*. [16], who also reported similar outcomes for UFP and AFP compared to well-adapted prefabricated fiber posts (PFP). Conversely, Maia *et al*. [20] reported that milled fiber posts performed worse than adapted PFP under severe weakening conditions but similarly under moderate weakening. Additionally, AFP combined with bulk-fill composite outperformed adapted PFP. Differences in study methodology, such as the absence of a ferrule in their study compared to the 2-mm ferrule present in this investigation, could explain these discrepancies. A ferrule thickness of at least 1 mm is crucial for improved resistance to functional forces, mitigating the wedge effect of conical posts and lateral stresses during post insertion [21,22].

Among the tested fiber posts, both UFP and MFP showed superior performance compared to AFP, consistent with previous studies [11,16,23]. These systems demonstrated excellent mechanical performance and are suiTable for clinical applications due to their enhanced fit and reduced cement line thickness, improving mechanical retention [24], regardless of the level of root weakening observed in this study.

The anatomic post technique involves the addition of adhesive interfaces between the fiber post, composite resin, and cement, which increases technique sensitivity and chairside time [25]. Although relining posts with composite resin is common, using a fiber sleeve or a single adjusTable post provides a simpler alternative [16]. The UFP system’s superior performance can be attributed to stress distribution facilitated by the sleeve and internal cementation line [16].

MFP offers greater precision, better fit, and improved sliding frictional retention within the root canal [23]. For flared canals, MFP reduces cement thickness and achieves better adaptation, optimizing stress distribution and increasing fracture resistance [11,23]. Proper fiber orientation during manufacturing is critical for the mechanical performance of glass fiber posts. Unidirectional fibers provide higher strength when stress is parallel to the fiber direction, but strength decreases under perpendicular forces [27]. In this study, unidirectional fibers in the MFP group contributed to the favorable results, emphasizing the importance of vertical fiber alignment during milling [27].

Fracture patterns varied between post systems. UFP and MFP exclusively resulted in repairable fractures, regardless of the weakening protocol, while AFP showed both repairable and irreparable fractures under severe weakening conditions. This difference is likely due to variations in the modulus of elasticity among the materials. The modulus of elasticity for MFP (25 GPa) closely resembles that of dentin (17.06–25.07 GPa), providing better stress distribution, whereas the modulus for prefabricated posts and UFP (40 GPa) and bulk-fill composite resin (10–12 GPa) differ significantly [28]. The control group exhibited only repairable fractures, supporting the notion that the presence of a ferrule enhances fracture repairability and reduces catastrophic failures [21].

The study also assessed the performance of monolithic polycrystalline tetragonal zirconia crowns (5Y-TZP). This material, known for its high translucency and mechanical properties, is ideal for anterior restorations and posterior bridges of up to three units. Although 5Y-TZP has superior mechanical properties compared to lithium disilicate, its esthetics are comparatively lower [29,30].

The findings suggest that MFP and UFP are effective for restoring flared root canals, outperforming the anatomic post technique. Their superior internal adaptation, reduced cement thickness, and effective force distribution contribute to favorable outcomes with less technique sensitivity. However, the MFP technique incurs higher costs and requires laboratory fabrication, which may limit its clinical application.

This study simulated a static compressive load, which does not fully replicate the dynamic forces present in clinical scenarios. Further randomized clinical trials are required to validate these findings and assess the long-term clinical performance of these restorative techniques.

## Conclusions

CAD/CAM milled and universal 2-piece fiber posts demonstrated favorable performance in restoring weakened roots, serving as a viable alternative. The weakened protocol influenced the performance of all fiber post types. Repairable failure modes predominated across all restorative strategies.

## Figures and Tables

**Table 1:** Description of the groups.

Groups (n=10)		Description
Weakened Protocol	Restorative Strategy	
Non-Weakened (NW)	Prefabricated Fiber Post	Prefabricated Fiber Posts (Exacto n° 2, Angelus) were cemented in the non-weakened roots, simulating an adequate adaptation of the post to the post space.
Moderate Weakened (MW)	Anatomical Fiber Post	Moderate weakened roots, restored with Prefabricated Fiber Posts (Exacto n° 0.5, Angelus) relined with Bulk Fill resin composite (Filtek One Bulk Fill, 3M ESPE)
	CAD/CAM Milled Fiber Post	Moderate weakened roots, restored with Milled Fiber Posts (Fiber Cad Post&Core, Angelus)
	Universal 2-Piece Fiber Post	Moderate weakened roots, restored with Universal 2-Piece Fiber Post (Splendor Universal, Angelus)
Severe Weakened (SW)	Anatomical Fiber Post	Severe weakened roots, restored with Prefabricated Fiber Posts (Exacto n° 0.5, Angelus) relined with Bulk Fill resin composite (Filtek One Bulk Fill, 3M ESPE)
	CAD/CAM Milled Fiber Post	Severe weakened roots, restored with Milled Fiber Posts (Fiber Cad Post&Core, Angelus)
	Universal 2-Piece Fiber Post	Severe weakened roots, restored with Universal 2-Piece Fiber Post (Splendor Universal, Angelus)

**Table 2:** Composition of the restorative materials used in this study.

Material	Manufacture	Composition
Exacto ® Glass Fiber Post #0.5	Angelus, Londrina, PR, Brasil	Glass Fiber (80%) and epoxy resin (20%)
Exacto ® Glass Fiber Post #2	Angelus, Londrina, PR, Brasil	Glass Fiber (80%) and epoxy resin (20%)
Splendor ® Universal Glass Fiber Post	Angelus, Londrina, PR, Brasil	Glass Fiber (80%) and epoxy resin (20%)
Fiber CAD Post&Core ®	Angelus, Londrina, PR, Brasil	Glass Fiber (75-80%) and epoxy resin (20-25%)
Monobond N	Ivoclar Vivadent, Schaan, Liechtenstein	Alcohol solution of silane methacrylate, phosphoric acid methacrylate and sulphide methacrylate
Single Bond Universal	Solventum, Sumare, SP, Brazil	Ethanol-based solvent: 10-MDP; phosphate monomer; dimethacrylate resin; HEMA; methacrylate modified polyacenoic acid copolymer; ethanol; water; primers; silane
Filtek One Bulk Fill	Solventum, Sumare, SP, Brazil	AFM; AUDMA; UDMA; 1,12-dodecane-DMA; 20-nm silica, 4-11 nm zirconia; 100 nm agglomerated ytterbium trifluoride particles
RelyX U200	Solventum, St. Paul, MN, USA	Base paste: methacrylate monomers containing phosphoric acid groups, methacrylate monomers, silanated fillers, initiator components, stabilizers, rheological additives. Catalyst paste: methacrylate monomers, alkaline (basic) fillers, silanated fillers, initiator components, stabilizers, pigments and rheological additives.

**Table 3:** Mean and standard deviation of the fracture resistance values (N) according to the weakened protocol and restorative strategy.

RESTORATIVE STRATEGY	WEAKENED PROTOCOL		
	Moderate Weakened	Severe Weakened	Main Factor Restorative Strategy
Anatomic fiber post	1043.0±109.4	996.7±128.9	1019.9±32.7 B
CAD/CAM milled fiber post	1301.5±113.7	1194.2±109.3	1247.9±75.9 A
Universal 2-piece fiber post	1199.2±154.7	1128.6±125.5	1163.9±49.9 A
Main Factor Weakened protocol	1181.2±130.2 a	1106.5±100.6 b	

* Different letters indicate a significant difference between the values (*p* <0.05) for the main factors weakened protocol (lower case) and restorative strategy (upper case), using a 2-way ANOVA and Tukey post hoc test.

**Table 4:** Comparison of fracture strength values (N) between control and experimental groups using Student’s t-test for independent samples.

WEAKENED PROTOCOL	NON-Weakened (control)	Moderate Weakened			Severe Weakened		
Restorative Strategy	PFP (CONTROL)	AFP	MFP	UFP	AFP	MFP	UFP
Fracture Resistance	1077.0±121.5	1043.0±109.4	1301.5±113.7	1199.2±154.7	996.7±128.9	1194.2±109.3	1128.6±125.5
Control*Experimental	-	p=0.565	p=0.002	p=0.101	p=0.220	p=0.062	p=0.418

PFP: Prefabricated fiber post; AFP: Anatomical fiber post; UFP: universal fiber post; MFP: CAD/CAM milled fiber post ǂ statically different from control group

**Table 5:** Failure mode in each group (n=10) concerning the factors restorative strategy and weakened protocol and frequency (%) of catastrophic failure.

WEAKENED PROTOCOL *	RESTORATIVE STRATEGY	FAILURE MODE		FREQUENCY OF CATASTROPHIC (%)
		REPAIRABLE	IRREPARABLE	
NON-WEAKENED	PFP	10	0	0
MODERATE WEAKENED	AFP	9	1	10
	UFP	10	0	0
	MFP	10	0	0
SEVERE WEAKENED	AFP	9	1	10
	UFP	10	0	0
	MFP	10	0	0
TOTAL		68	2	2.86

* *p* value=0.842 by chi-square test (weakened protocol× failure mode) ǂ *p* value=0.161 by chi-square test (restorative strategy × failure mode)

## Data Availability

The datasets used and/or analyzed during the current study are available from the corresponding author.
